# Zinc and Copper Oxide Nanoparticles: Pioneering Antibacterial and Antibiofilm Strategies for Environmental Restoration against Antibiotic-Resistant Bacteria

**DOI:** 10.3390/ma17143444

**Published:** 2024-07-12

**Authors:** Chandrabose Uthra, Karuppiah Nagaraj, Mohammad Ahmad Wadaan, Chelladurai Karuppiah, Prasenjit Maity, Almohannad Baabbad, Raja Kaliyaperumal, Renuka Venkatachalapathy, Flora Shah, Puneet Kumar

**Affiliations:** 1 Department of Microbiology, Bharathidasan University, Tiruchirappalli 620024, Tamil Nadu, India; uthra027@gmail.com; 2 School of Pharmacy, National Forensic Sciences University, 6M56+XP8, Police Bhavan Rd, Sector 9, Gandhinagar 382007, Gujarat, India; 3 Department of Zoology, College of Science, King Saud University, P.O. Box 2455, Riyadh 11541, Saudi Arabia; 4 Battery Research Center of Green Energy, Ming Chi University of Technology, New Taipei City 243303, Taiwan; 5 Center of Molecular Medicine and Dianostics (COMManD), Saveetha Dental College and Hospitals, SIMTS, Saveetha University, Chennai 600077, Tamil Nadu, India; 6 School of Environmental Technology, National Forensic Sciences University, 6M56+XP8, Police Bhavan Rd, Sector 9, Gandhinagar 382007, Gujarat, India; 7 Department of Chemistry, St. Joseph University, Chumoukedima 797115, Nagaland, India

**Keywords:** wastewater treatment, antibiotic resistant bacteria, nanoparticles, antibacterial, copper oxide, zinc oxide, antibiofilm

## Abstract

This study explores the challenge of antimicrobial resistance by investigating the utilization of zinc oxide (ZnO) and copper oxide (Cu_2_O) nanoparticles (NPs) to combat antibiotic-resistant bacteria in wastewater treatment plants (WWTPs). The synthesized metal oxide NPs underwent thorough characterization through various analytical techniques, confirming their nanoparticulate nature. Electronic absorption and X-ray diffraction (XRD) analyses revealed successful reduction processes and crystalline properties, respectively. Fourier transform infrared spectroscopy (FTIR) results indicated the stabilization of nanoparticles in solution. Scanning electron microscopy (SEM) observations revealed well-defined spherical and flower-like morphologies for the zinc and copper oxide nanoparticles, with sizes approximately ranging from 50 nm to 25 nm Notably, the synthesized nanoparticles exhibited heightened efficacy in impeding biofilm formation, with zinc oxide NPs displaying superior antibacterial activity compared to copper. These findings suggest the promising potential of these nanoparticles in controlling antibiotic-resistant organisms, even following WWTP treatment processes. This research contributes to the ongoing advancements in nanotechnology aimed at combating antibiotic resistance, offering new prospects for the development of effective wastewater treatment strategies.

## 1. Introduction

In recent decades, the increasing demand for fresh water, driven by population growth, urbanization, and industrialization, has led to a rise in water pollution. This poses a significant threat to the natural environment and the survival of living organisms. The volume of effluents containing rich organic constituents from industrial sites as well as from urban agglomerations are being increased frequently, mainly from undeveloped and developing countries [1]. Most countries rely heavily on groundwater resources for their water supply [2]. Although natural purification processes exist, they are slow and not always effective in significantly purifying wastewater. Its discharge into natural water bodies poses a significant concern for maintaining a sustainable environment conducive to human welfare [3,4]. Additionally, wastewater treatment plants (WWTPs) play a crucial role in reducing freshwater ecosystem degradation, aligning with water management frameworks [5,6]. The previous literature has highlighted favorable conditions within WWTPs for the proliferation and transmission of antibiotic-resistant bacteria (ARB) to non-resistant strains [7,8,9,10]. It is imperative to identify the abundance and tolerance of ARB and antibiotic resistance genes (ARGs) in WWTPs for effective management. Various mobile genetic elements, including plasmids, transposons, bacteriophages, integrons, and their combinations, facilitate the transfer of antibiotic resistance within wastewater treatment plants (WWTPs) and other environmental settings. [11,12,13,14,15]. The unique characteristics and extensive range of uses of zinc oxide nanoparticles (ZnO NPs) and cuprous oxide nanoparticles (Cu_2_O NPs) make them noteworthy. Due to their antibacterial and anticancer properties, ZnO NPs, which have a wurtzite crystal structure, are suited for usage in optoelectronics, sensors, and biomedical applications [16]. They also have a broad bandgap of 3.37 eV, high electron mobility, and strong UV absorption. Their size and shape may be precisely controlled using a variety of synthesis techniques, including hydrothermal, green, and sol-gel. Conversely, visible light is absorbed by Cu_2_O nanoparticles (NPs), which have a bandgap of approximately 2.0 eV and a cuprite crystal structure. This allows for their implementation in solar cells, photocatalysis, and antimicrobial applications [17].

Zinc and copper oxide NPs are of great interest due to their unique properties and versatile applications. ZnO NPs exhibit excellent UV absorption capabilities, antimicrobial properties, and semiconductor behavior, making them valuable in sunscreen formulations, antibacterial coatings, optoelectronic devices, and biomedical applications. On the other hand, Cu_2_O NPs possess significant photocatalytic activity, good conductivity, and versatile catalytic properties, rendering them useful for environmental remediation, energy conversion, electronic devices, and biomedical applications. These metal oxide NPs hold promise for addressing various challenges in fields ranging from healthcare and environmental science to energy and electronics.

Biofilms, which form in various habitats through natural or artificial processes, consist of a complex matrix of biopolymers. These biofilms enhance survival and resistance, shielding organisms from environmental hazards [18,19,20,21,22,23]. This reduced susceptibility is caused by a combination of different factors that act synergistically with those responsible for conventional resistance linked to the presence of ARGs, yielding an overall increased resistance of biofilms to antimicrobial compounds.

In recent years, there has been growing research interest in the application of transition-metal-based photocatalytic materials and their interaction with biofilm formation. These materials have shown potential in various catalytic and sensor applications [24,25,26,27,28,29]. *Acinetobacter* sp., *Escherichia coli*, *Klebsiella* sp., *Staphylococcus aureus*, *Bacillus megaterium*, and *Thiobacillus aquaesulis* are among the most common biofilm-forming bacteria responsible for human diseases such as infectious lesions in endocarditis, cystic fibrosis, and otitis media with effusion. Such microorganisms develop drug resistance by various mechanisms; biofilm formation is one of such central mechanisms. In contrasting the treatment of biofilms with antibiotics to the conventional methods, the nanotechnology-based approach stands out as an efficient method to combat biofilm formation [16,17,30]. The use of nanoparticles is considered a viable solution for preventing infectious diseases due to their antimicrobial properties. The present work demonstrates that zinc and copper oxide nanoparticles can be used to control ARB in WWTPs and the spread of resistance in the WWTP’s surrounding environment. Due to the significant problem of advancing antimicrobial resistance, the scientific community has attempted to find alternative solutions and one of the most promising attempts is to use metal nanoparticles.

## 2. Materials and Methods

### 2.1. Materials

All chemicals, including zinc sulfate (purity: >99%), sodium hydroxide (purity: >98%), cupric sulfate (purity: >99%), potassium sodium tartrate tetrahydrate (purity: >98%), glucose (purity: >99%), and ethanol (purity: >99.5%), were procured from Hi-media Laboratory, Mumbai, India. The solvents and reagents employed were of analytical grade, while all glassware utilized throughout the study was sourced from Borosil, Mumbai, India. PCR primers targeting the 16S rRNA gene were acquired from VBC-Biotech Eurofins Genomics, Vienna, Austria.

### 2.2. Methods

The research methods employed state-of-the-art equipment for various analyses. Weighing was conducted using a top-loading mono-pan electronic balance (Shimadzu, Kyoto, Japan). UV–Vis spectroscopy measurements were performed using a UV–Vis spectrophotometer (UV-1700, Shimadzu, Japan). The separation of absorbents and protein samples was achieved using a refrigerated centrifuge (SIGMA-3K 30 ScQuip, Newtown, UK), while the morphological characterization of microorganisms was carried out using a microscope (Olympus-CH20i, Gurugram, India). Glycerol stocks of bacterial cultures, bacterial protein, and DNA were stored in a deep freezer (Siemens KG-57, Mumbai, India) at −85 °C. Microbial culturing and incubation were conducted using a shaker (Orbitek9 Scignics Biotech, Chennai, India) and an incubator (KEMI KLS.2, Mudickal, India), respectively. DNA and protein separation and analysis were performed using a vertical and horizontal model electrophoresis unit (Bio-rad, Neuried, Germany) and a power pack (PowerPC TM basic power supply-Biorad, Neuried, Germany) with constant voltage/constant current. Gel documentation was performed using a gel documentation unit (Biorad XR, Neuried, Germany), while pure bacterial cultures were obtained using a laminar airflow chamber (Kleanzone SP0.82, Chennai, India). DNA amplification was carried out using a thermal cycler (Bio rad T-100, Neuried, Germany), and microbial growth analysis was conducted using an ELISA reader (ELx800, BioTek, Oviedo, FL, USA). Nucleotide sequence comparison was performed with Blast software, (BLAST + 2.6.0), 2017, National Centre for Biotechnology Information (NCBI). X-ray diffraction (XRD) analysis was conducted using an X-ray diffractometer with Kα radiation, and Fourier transform infrared spectroscopy (FTIR) analysis was performed using a Shimadzu IR Prestige-21 instrument. Finally, scanning electron microscopy (LEO 1430 VP, Carl Zeiss AG, Oberkochen, Germany) was used to characterize the surface structure and morphology of nanoparticles, with samples coated in carbon-coated ZnO and Cu_2_O grids. For dynamic light scattering (DLS) analyses, samples were prepared by dispersing the nanoparticles in a suitable solvent at a concentration within the linear range of the instrument. The DLS analyzer used in this study was the Zetasizer Nano ZS (Malvern Panalytical, Malvern, UK), equipped with a 4 mW He-Ne laser operating at a wavelength of 633 nm. Care was taken to avoid introducing air bubbles during the dispersion process, as they can interfere with the accuracy of the DLS measurements.

### 2.3. Synthesis of NPs

#### 2.3.1. Zinc Oxide Nanoparticle Synthesis

To synthesize zinc oxide (ZnO) nanoparticles, a zinc salt precursor such as ZnSO_4_·7H_2_O (0.05 M) is dissolved in water to form a clear solution. Subsequently, a base NaOH (0.2 M) was added dropwise to the solution under constant stirring until the pH reached the desired value, typically around 10–12, inducing precipitation, and the reaction mixture was then heated to a moderate temperature of 80–100 °C under continuous stirring for several hours to facilitate the ZnO nanoparticles. Following this, the resulting precipitate was washed multiple times with a suitable solvent, such as water or ethanol, to remove impurities and unreacted precursors. Finally, the washed precipitate was dried at a low temperature, below 100 °C, to obtain the final ZnO nanoparticles [31].

#### 2.3.2. Copper Oxide Nanoparticles Synthesis

**Scheme 1:**
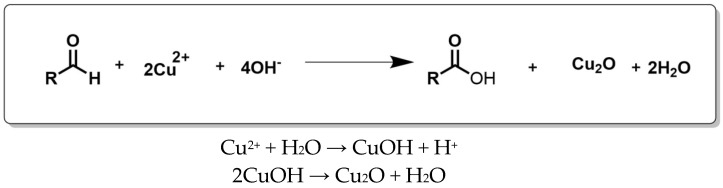
Mechanism of chemical reactions.

In this procedure, 6.9 g of copper sulfate was dissolved in 100 mL of deionized water. To this solution, 34.6 g of potassium sodium tartrate tetrahydrate and 12 g of sodium hydroxide were added. The mixture was vigorously stirred for 15 min. Additionally, 5 g of glucose in 50 mL of water was prepared and added to the solution, which was then heated to 60 °C under continuous stirring. Following synthesis, the resulting material was purified by washing three times with double-distilled water and twice with 100% ethanol. Subsequently, it was air-dried for 3 h in a hot-air oven. The reduction of Cu^2+^ to Cu^+^ played a pivotal role, leading to the generation of Cu_2_O through the hydrolysis of Cu^+^, as described by the following reactions (Scheme 1) [32].

### 2.4. Isolation of Bacteria

Bacteria were isolated by suspending 1 mL of collected water samples in 9 mL of autoclaved distilled water, followed by serial dilution and inoculation on agar medium. The plates were then incubated at 37 °C until bacterial colonies appeared on NA medium. Visibly distinguishable bacterial colonies were selected, and the colony morphology of each isolate was recorded. The selected organisms were streaked onto nutrient agar (NA) plates to isolate pure cultures and were maintained under freeze-drying conditions and stored in glycerol at 4 °C.

### 2.5. DNA Isolation

About 1.5 mL of overnight culture was centrifuged for 5 min. The pellet was taken, and the supernatant was discarded. To the pellet, 567 µL of TE and 10 µL of RNase were added, which were mixed well and incubated at 37 °C for 1 h. Then, 30 µL of 10% SDS and 3 µL of proteinase K was added and kept in a water bath at 37 °C for 1 h. After the completion of incubation time, 100 µL of 5 M NaCl and 80 µL of CTAB/NaCl were added and kept at 65 °C in a water bath for 10 min. After the incubation, 500 µL of phenol:chloroform:isoimylalcohocal (25:24:1) was added and centrifuged at a maximum speed of 5 min. Then, the upper layer of the aqueous phase (from 3 layers) was transferred into a new Eppendorf tube with 0.6 mL of isopropanol, which was then tilted well. A resulting thread-like structure indicated the presence of DNA. After centrifuging at maximum speed for 5 min, the supernatant was discarded. After the pellet was inverted and air dried for 5 to 10 min, 500 µL of 70% ethanol was poured onto the tissue paper (tubes opened). They were centrifuged for 5 min, then dried again for a few minutes, and then a mixture of 50 µL of TE buffer was added (to dissolve the DNA), followed by storage in a refrigerator at −20 °C.

### 2.6. DNA Quantification

The quantity of DNA was determined by measuring the ratio of OD at 260 nm and 280 nm using a UV–Vis spectrophotometer. DNA quantification was calculated as per the following formula:

Quantity of DNA (ng/µL) = (260 nm/280 nm)

### 2.7. Agarose Gel Electrophoresis

Agarose gel (0.85%) was prepared in 1× TAE electrophoresis buffer, to which 4 µL of ethidium bromide (10 mg/mL) was added. A loading buffer (10×) was added to DNA samples for separation. Samples and DNA molecular weight markers were loaded. The samples were run for 2 h at 50 V (Bio-rad) and DNA was visualized under UV light on a gel documentation system (Bio-rad T100 TM Thermal Cycler).

### 2.8. PCR Amplification for 16S rRNA

The extracted DNA was amplified to obtain near-full-length 16S rRNA genes by polymerase chain reaction (PCR) (Table 1).

### 2.9. Agarose Gel Electrophoresis of PCR Product

A PCR-amplified product aliquot of about 5 µL was electrophoresed on a 1.4% agarose gel in 1XTAE buffer at 50 V for 45 min. It was stained with ethidium bromide and the PCR product was visualized under UV light on a gel documentation system.

## 3. Results

### 3.1. Morphological and Physiological Studies

In this work, the resistance bacterial diversity associated with municipal wastewater treatment plants was examined using a culture-dependent technique. Bacterial growth was observed on nutrient agar plates after the incubation of 24–48 h. The water samples (Supplementary Table S1) were collected in a wastewater treatment plant (WWTP) located in Pudukkottai, Tamil Nadu, India. In total, two samples were collected and named T1 (raw sewage water collected from the primary inlet) and T2 (treated water). The samples were collected in a sterile container by placing the container in the opposite direction to the water current at a depth of 5 to 10 cm. Samples were collected, immediately transported to the lab, and microbial isolation was completed in less than a day.

### 3.2. Studies of rRNA Sequence Analysis

The banding pattern of DNA samples isolated from 27 organisms indicated that the genomic DNA molecular weight was 20,000 kb. About 0.1 ng and 28 cycles were required for amplification. Distinct PCR products of the 1500 pb (Supplementary Figure S1) size were produced from the target DNA isolated from 27 bacterial strains.

### 3.3. Antibiotic Resistance Profile

**Figure 1:**
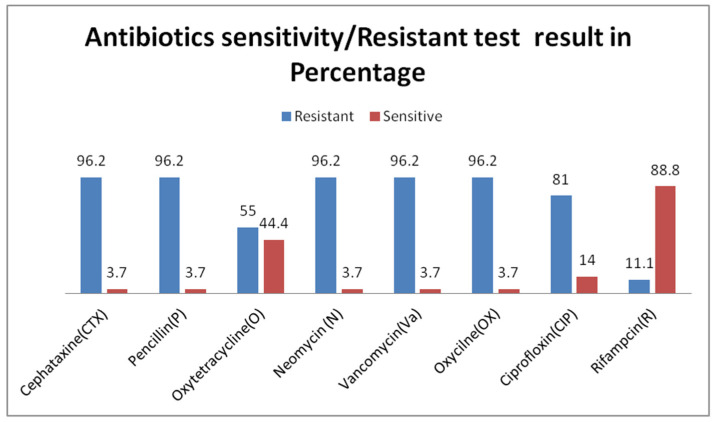
Antibiotic resistance profiles of isolated organisms.

Using the antibiotic disc diffusion method, in vitro antibiotic susceptibility testing was carried out on 27 isolated bacterial strains. Out of the eight drugs examined, all bacteria showed multidrug resistance to six or seven of them. The resistance patterns were as follows: Cefotaxime (96%), Penicillin G (96%), Oxytetracycline (55%), Neomycin (96%), Vancomycin (96%), Oxacillin (96%), Ciprofloxacin (81%), and Rifampicin (11.1%). The resistance profiles were determined using the Kirby Bauer disk diffusion method, employing antibiotic discs on Mueller–Hinton agar plates (Figure 1). The results were interpreted as susceptible or resistant based on criteria outlined by the Clinical Laboratory Standards Institute (CLSI, 2005). 

### 3.4. Biofilm Formation Assay

The biofilm quantification was performed using a microplate assay, as depicted in Supplementary Figure S3. Optical density (OD) readings were obtained at wavelengths of 600 nm and 570 nm using an ELISA reader. The mean OD values at 600 nm were subtracted from the mean OD values at 570 nm for the test strains. Based on the resulting biofilm index values, the isolates were categorized as biofilm-forming or non-biofilm-forming organisms [33], as described by Stepanovic et al. Out of the 27 tested organisms, 6 were identified as strong biofilm formers.

### 3.5. Characterization of Metal Oxide NPs

**Figure 2:**
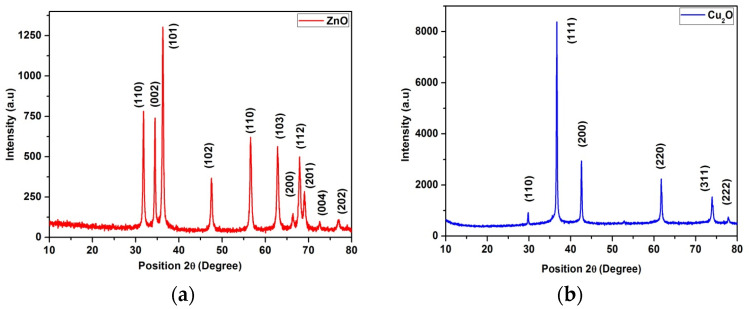
(**a**) XRD pattern of ZnO; (**b**) Cu_2_O.

The electronic absorption spectrum of zinc and copper oxide NPs showed a maximum intensity at 369 nm and above 500 nm, as depicted in Supplementary Figure S5a,b. Additionally, the IR spectrum (Supplementary Figure S6a,b) revealed the respective functional moieties. For the preliminary identification of the synthesis of zinc oxide and cuprous oxide nanoparticles, XRD analyses were carried out. All the peaks were indexed using the JCPDS card no: 00-001-1136 (ZnO) and 03-065-3288 (Cu_2_O), and no impurities of the precursors were observed (Figure 2a,b). Dynamic light scattering is a quick, appropriate, and definite method that was used for the measurement of the synthesized copper and zinc oxide nanoparticles in suspension, as shown in Supplementary Figure S5a,b. We calculated the crystallite sizes of ZnO and Cu_2_O based on the XRD results using the Scherrer equation:

*D* = *Kλ/β*cos*θ*

where:

*D* is the average crystallite size,
*K* is the Scherrer constant (typically taken as 0.9),
*λ* is the wavelength of the X-ray radiation used (in Å),
*β* is the full width at half maximum of the peak (in radians),
*θ* is the Bragg angle (in radians).

Once the FWHM (*β*) and Bragg angle (*θ*) are determined from the XRD data, they can be substituted into the equation along with the appropriate values of *K* and *λ* for the X-ray source used. The estimated mean crystallite sizes were of ZnO and Cu_2_O nanoparticles with sizes ranging from approximately ~50 nm to ~25 nm, respectively.

**Figure 3:**
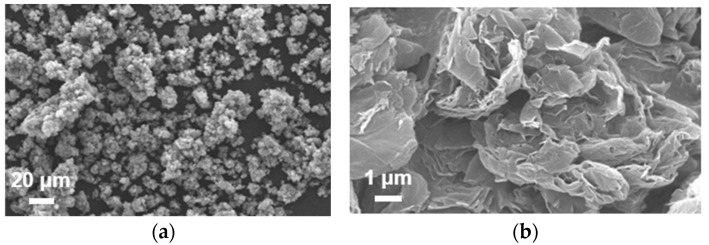
(**a**) SEM image of ZnO. (**b**) SEM image of Cu_2_O.

SEM visualization allows for the measurement of both the size and shape of the metal oxide nanoparticles formed. The SEM images of the zinc and copper oxide nanoparticles are presented in Figure 3a and b, respectively. The zinc nanoparticles appeared spherical in shape, exhibiting a wide distribution of sizes in the range of approximately 50 nm. In contrast, the copper nanoparticles displayed a flower-like shape, with a similar wide distribution of sizes ranging around ~25 nm.

### 3.6. Antibacterial and Antibiofilm Activity

**Figure 4:**
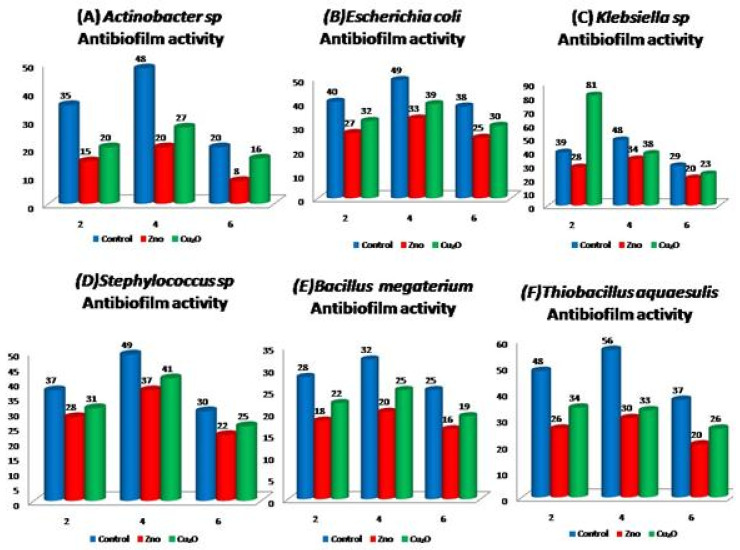
(**A**–**F**) Antibiofilm activity using different specimens.

Mueller–Hinton broth (MHB, 150 µL) was added to each well of the sterile 96-well plates. The zinc oxide and copper oxide nanoparticles were diluted in distilled water (1 mg/mL) the first five concentrations of the two-fold dilution were taken [34]. The blank consisted of only the media. The positive control included the media and the first dilution of the 50µL of nanoparticle suspension. For the negative control, the media and 50 µL of bacterial broth culture were used. The media (150 µL), culture (50 µL), and 50 µL of different concentrations of nanoparticles were added to each well. The microtiter plates were covered and incubated at 37 °C for 24 h. After the incubation time, the plates were read on the ELISA plate reader at 600 nm. Biofilm inhibition by the zinc oxide and copper oxide NPs was examined based on the ELISA plate reader OD values, and the highest biofilm inhibition nanoparticles concentration was identified. The biological activity of the zinc and copper oxide NPs was tested at five concentrations against the six highly biofilm-producing multidrug-resistant organisms tested, namely *Acinetobacter* sp., *Escherichia coli*, *Klebsiella* sp., *Staphylococcus aureus*, *Bacillus megaterium*, and *Thiobacillus aquaesulis*. The results showed that zinc had a high level of antibacterial activity compared to the copper oxide NPs. All the nanoparticles tested showed reduced bacterial growth, and the rate of reduction depended on the time and concentration used, as shown in Supplementary Figure S8a,b. The antibiofilm activity of the synthesized zinc- and copper-oxide-nanoparticle-coated glass specimens were assayed with the six strains capable of producing a strong biofilm, namely *Acinetobacter* sp., *Escherichia coli*, *Klebsiella* sp., *Staphylococcus aureus*, *Bacillus megaterium*, and *Thiobacillus aquaesulis* (Figure 4).

## 4. Discussion

In this work, the diversity of bacterial resistance associated with municipal wastewater treatment plants was examined using a culture-dependent technique. Bacterial growth was observed on nutrient agar plates after incubation for 24–48 h. In total, 27 morphologically different colonies were isolated from WWTPs. Among them, 18 colonies were found in T1 (raw sewage water), and 9 colonies were found in T2 (treated sewage water). All 27 bacterial strains were identified at their generic level according to Bergey’s manual [35,36], which is shown in Supplementary Table S1. These identified strains were employed for antibiotic resistance studies. Distinct PCR products of 1500 pb size were produced from target DNA isolated from the 27 bacterial strains as shown in Supplementary Figure S1. The amplified products were subjected to 16S rRNA gene sequence analysis to identify the species of isolated bacterial strains. The partial sequencing of 16S rRNA resulted in different lengths that varied for each of the 27 organisms. The amplified products were supported by other organisms, with a specific bootstrap value for each isolated organism. Higher bootstrap values are indicated in the tree shown in Supplementary Figure S2. Isolated bacterial strains (27) were tested in vitro to determine their antibiotic susceptibility pattern by the antibiotic disc diffusion method (Supplementary Figure S3). All the strains showed multidrug resistance to six or seven antibiotics among the eight antibiotics tested. The resistance patterns were as follows: Cefotaxime (96%), Penicillin G (96%), Oxytetracycline (55%), Neomycin (96%), Vancomycin (96%), Oxacillin (96%), Ciprofloxacin (81%), and Rifampicin (11.1%). The resistance ability was measured by the Kirby method with antibiotic discs on Mueller–Hinton agar plates as shown in Figure 1. The quantitative estimation of the biofilm was made by the microtiter plate assay (Supplementary Figure S4). Optical density (OD) was recorded at 600 and 570 nm using an ELISA reader. The mean values of OD 600 nm were subtracted from the mean values of OD 570 nm of the test strains. Based on the biofilm index value, the isolates were then identified as biofilm-forming or non-biofilm-forming organisms [3]. Among the 27 tested, 6 organisms were selected as strong biofilm-forming organisms. Electronic absorption was performed for the synthesized zinc and copper oxide nanoparticles [31,32]. The formation of a brown color clearly indicated the surface resonance and the deposition of an inter-band transition, particularly in terms of the size of the effect. The absorption spectrum of the medium containing zinc and copper showed a maximum intensity at 369 nm and above 500 nm, as shown in Supplementary Figure S5a,b. For the preliminary identification of the synthesis of the zinc oxide and cuprous oxide nanoparticles, XRD analyses were carried out. All the peaks were indexed using the JCPDS card no: 00-001-1136 (ZnO) and 03-065-3288 (Cu_2_O), and no impurities of the precursors were observed. This showed that the prepared sample was highly pure. The sharp peak in both the samples indicated the crystalline nature of the material. In the case of ZnO, the sharp peak at (101) represents the wurtzite structure. The crystal lattice parameters were found to be a(Å) = 3.2420, b(Å) = 3.2420, and c(Å) = 5.1760. All the peaks were sharp, which showed that the material was highly oriented. Similar to ZnO, Cu_2_O also showed high crystallinity. Peaks at (111) confirmed the formation of the cubic phase of cuprous oxide. The lattice parameter values were found to be a(Å) = 4.2600, b(Å) = 4.2600, and c(Å) = 4.2600, as shown in Figure 2a,b. The FTIR spectrum of synthesized zinc nanoparticles showed strong bands at 3414, 1588.8, 1400.5, 898.14, and 559.07 cm^−1^. These bands correspond to primary amine N-H stretching, isothiocyanate (-NCS), the nitro functional group, and aliphatic bromo compound C-Br stretching, as shown in Supplementary Figure S6a. The copper oxide nanoparticles showed strong bands at 3418.1, 1605.6, 1396.3, and 617.7 cm^−1^. These bands correspond to alcohol/phenol O-H stretching, primary and secondary amine and amide N-H bending, and aliphatic amine functional group C-N stretching (Supplementary Figure S6b). The overall observation confirmed the presence of protein in the sample of zinc and copper oxide nanoparticles [37]. Dynamic light scattering is a quick, appropriate, and definite method that was used for the measurement of the synthesized copper and zinc oxide nanoparticles in suspension. The DLS analysis of the synthesized zinc nanoparticles yielded a nominal diameter of 266.8 nm with a standard deviation of 31.5 nm, whereas in the case of the synthesized copper oxide nanoparticles, a nominal diameter of 179.3 nm with a standard deviation of 13.8 nm was obtained as shown in Supplementary Figure S7a,b. The zinc nanoparticles were spherical in shape and showed a large distribution of sizes in the range of ~50 nm. The copper oxide nanoparticles showed a flower-like shape and a large distribution of sizes in the range of ~25 nm. The biological activity of the zinc and copper NPs were tested at five concentrations against the six highly biofilm-producing multidrug-resistant organisms tested, namely *Acinetobacter* sp., *Escherichia coli*, *Klebsiella* sp., *Staphylococcus aureus*, *Bacillus megaterium*, and *Thiobacillus aquaesulis*. The results showed that zinc had a high level of antibacterial activity compared to the copper oxide NPs. All the nanoparticles tested showed reduced bacterial growth, and the rate of the reduction depended on the time and concentrations of the zinc oxide (ZnO) and copper oxide (Cu_2_O) nanoparticles (NPs) at the various dilutions used, as shown in Supplementary Figure S8a,b. For ZnO, the concentrations ranged from 1 mg/mL for the first dilution to 0.06 mg/mL for the fifth dilution. Similarly, for Cu_2_O, the concentrations started at 1 mg/mL for the first dilution and decreased to 0.06 mg/mL for the fifth dilution. The antibiofilm activity of the synthesized zinc- and copper-oxide-nanoparticle-coated glass specimens were assayed using the six strains capable of producing a strong biofilm, namely *Acinetobacter* sp., *Escherichia coli*, *Klebsiella* sp., *Staphylococcus aureus*, *Bacillus megaterium*, and *Thiobacillus aquaesulis* (Figure 3). Figure 3 indicates that the cell survivability was lower for the zinc- and copper-oxide-nanoparticle-coated glasses compared to the control glasses, which corresponded to the results of the ICP analysis showing that the zinc and copper coatings were readily available to react with water and release ions. Therefore, we deduced that interactions either with DNA or with the proteins of the bacterial cell wall led to cell death, as revealed by the cell viability assay [38,39].

## 5. Conclusions

In conclusion, this study addresses the critical issue of antimicrobial resistance by exploring the application of zinc oxide (ZnO) and copper oxide (Cu_2_O) nanoparticles in combating antibiotic-resistant bacteria in wastewater treatment plants (WWTPs). The synthesized metal oxide NPs were characterized using a range of analytical techniques, including UV–Vis, FTIR, XRD, and DLS, affirming their nanoparticulate nature. The analysis revealed the reduction of zinc and copper ions, with distinctive electronic absorption peaks. The XRD results confirmed the crystalline nature of the nanoparticles, with sizes ranging around ~50 nm for zinc and ~25 nm for copper. FTIR indicated the stabilization of the nanoparticles in solution. Remarkably, the synthesized nanoparticles exhibited enhanced efficacy in inhibiting biofilm formation, with the zinc NPs demonstrating superior antibacterial activity compared to the copper oxide NPs. These findings underscore the promising potential of these nanoparticles in controlling antibiotic-resistant organisms, even post-WWTP treatment processes. Overall, this research contributes to the ongoing progress in nanotechnology for combating antibiotic resistance, offering novel avenues for the development of effective wastewater treatment strategies.

## Figures and Tables

**Table 1:** PCR primers for16S rRNA amplification.

Primer	Target for Amplification	Oligonucleotide 5′–3′
8F	16S rRNA	AGAGTTTGATCCTGGCTCAG
1492R	16S rRNA	TACGGCTACTTGTTACGACTT

## Data Availability

Data available made on request.

## Supplementary Materials

The following supporting information can be downloaded at: https://www.mdpi.com/article/10.3390/ma17143444/s1, Figure S1: PCR-amplified study of agarose gel electrophoresis; Figure S2: rRNA sequences of isolated organisms and other relative species; Figure S3: Antibiotic susceptibility pattern by antibiotic disc diffusion method; Figure S4: Biofilm formation assay; Figure S5: UV–Vis spectrum; Figure S6: FTIR spectrum; Figure S7: DLS results; Figure S8: Nanoparticle activity of different concentrations of ZnO and Cu_2_O; Table S1: Morphological and physiological characteristics of isolated bacterial strains.
