# Supplementary material for: Zinc and Copper Oxide Nanoparticles: Pioneering Antibacterial and Antibiofilm Strategies for Environmental Restoration against Antibiotic-Resistant Bacteria
Source: Materials (Basel). 2024 Jul 12;17(14):3444. doi: 10.3390/ma17143444 (PMC11278220; doi:10.3390/ma17143444)
Supplement: Supplementary file 1 [file materials-17-03444-s001.zip › materials-2954643-supplementary.pdf]

SUPPLEMENTARY INFORMATION

Zinc and Copper Oxide Nanoparticles: Pioneering Antibacterial and Antibiofilm Strategies for Environmental Restoration against Antibiotic-Resistant Bacteria

Chandrabose Uthra [1], Karuppiah Nagaraj [2,*], Mohammad Ahmad Wadaan [3], Chelladurai Karuppiah [4,5,*], Prasenjit Maity [6], Almohannad Baabbad [3], Raja Kaliyaperumal [7], Renuka Venkatachalapathy [6], Flora Shah [2] and Puneet Kumar [2]

[1] Department of Microbiology, Bharathidasan University, Tiruchirappalli 620024, Tamil Nadu, India;
uthra027@gmail.com

[2] School of Pharmacy, National Forensic Sciences University, 6M56+XP8, Police Bhavan Rd, Sector 9,
Gandhinagar 382007, Gujarat, India; florashah@gmail.com (F.S.); mypuneet11@gmail.com (P.K.)

[3] Department of Zoology, College of Science, King Saud University, P.O. Box 2455, Riyadh 11541, Saudi Arabia; wadaan@ksu.edu.sa (M.A.W.); almbaadbad@ksu.edu.sa (A.B.)

[4] Battery Research Center of Green Energy, Ming Chi University of Technology, New Taipei City 243303, Taiwan

[5] Center of Molecular Medicine and Dianostics (COMManD), Saveetha Dental College and Hospitals, SIMTS, Saveetha University, Chennai 600077, Tamil Nadu, India

[6] School of Environmental Technology, National Forensic Sciences University, 6M56+XP8, Police Bhavan Rd, Sector 9, Gandhinagar 382007, Gujarat, India; prasenjit.maity@nfsu.ac.in (P.M.);
renukavcbt@gmail.com (R.V.)

[7] Department of Chemistry, St. Joseph University, Chumoukedima 797115, Nagaland, India; krajaphd2012@gmail.com

* Correspondence: surfactantnagaraj@gmail.com (K.N.); kcdurai.rmd@gmail.com (C.K.); Tel.: +91-9944418072 (K.N.)

SUPPLEMENTARY INFORMATION

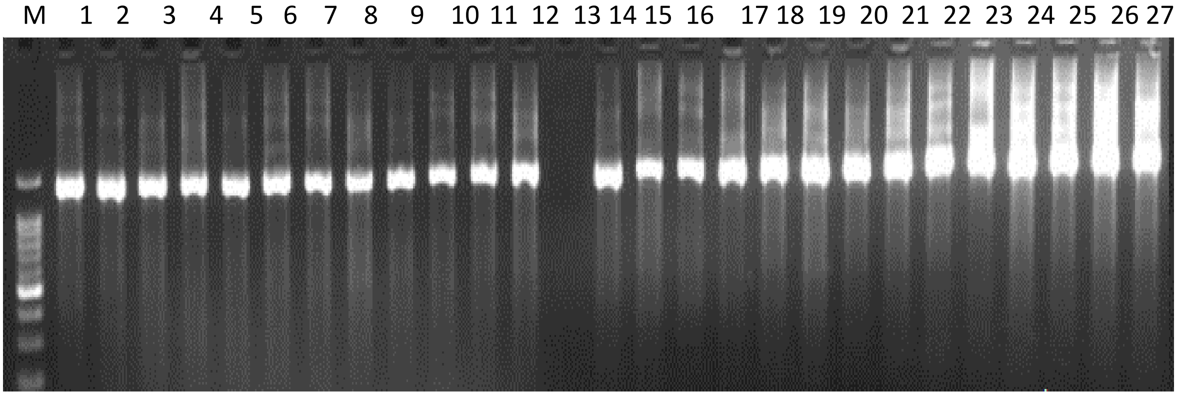

SI Figure S1. PCR amplified study of agarose gel electrophoresis (M- Marker; 1 to 27 – DNA samples of 27 isolated organisms).

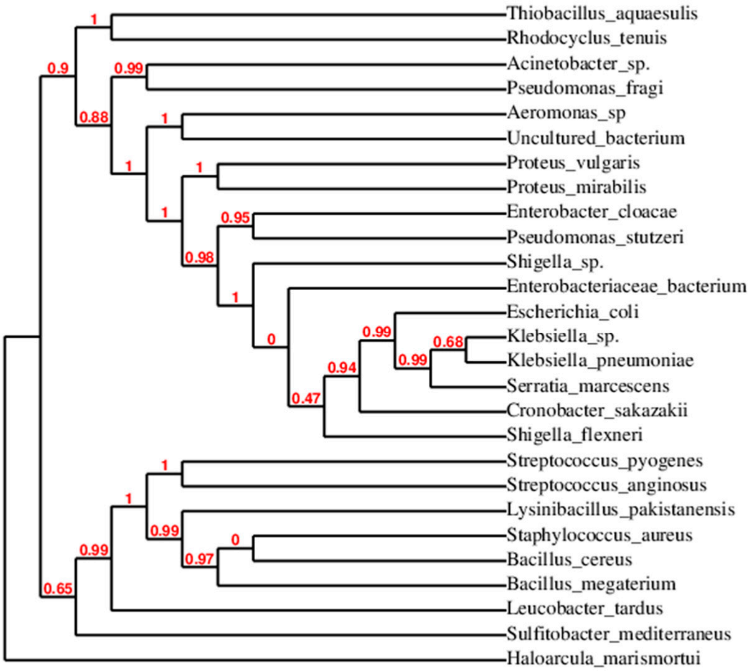

SI Figure S2. rRNA sequences of isolated organisms and other relative species

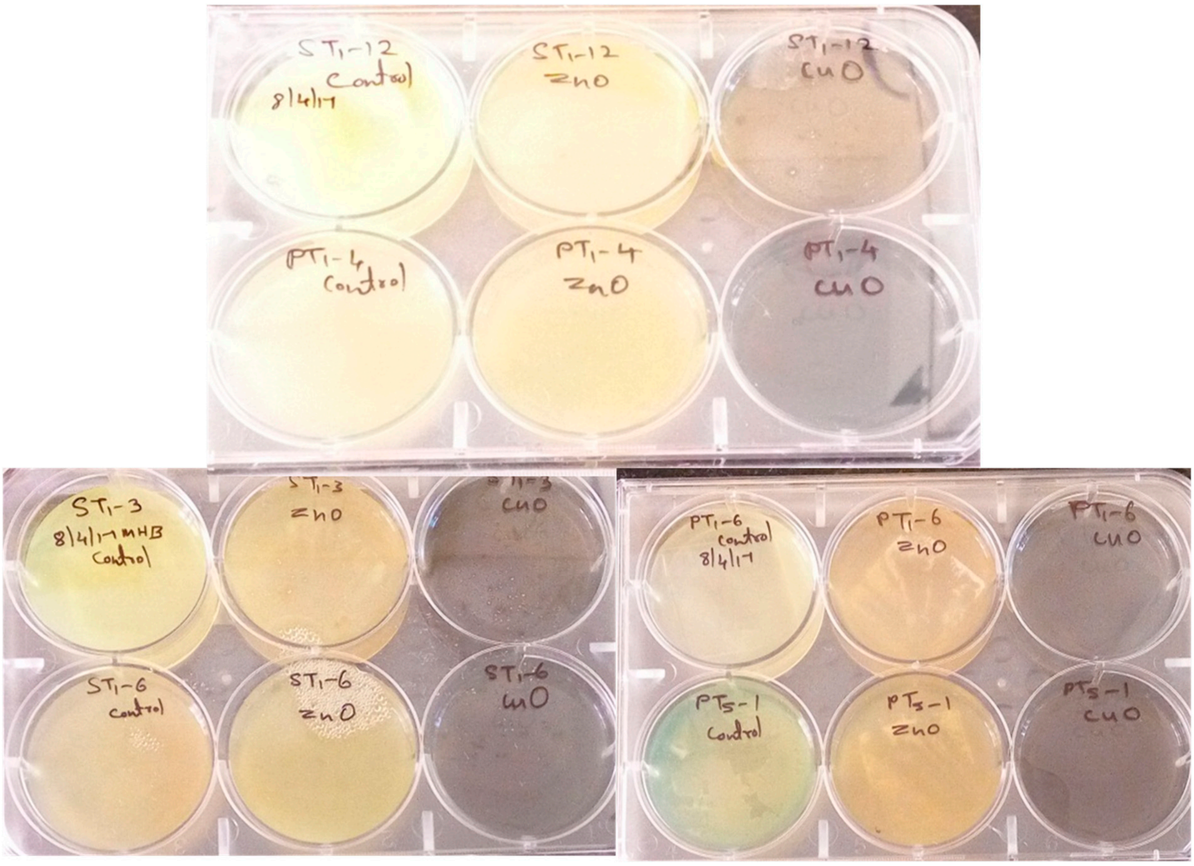

SI Figure S3. Antibiotic susceptibility pattern by antibiotic disc diffusion method.

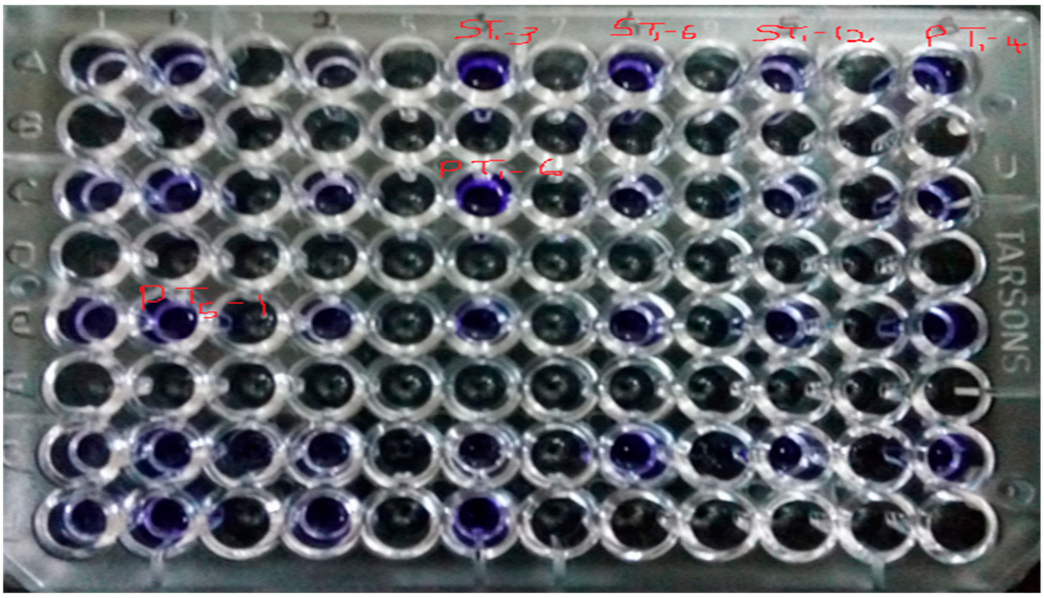

ST1-3 - *Acinetobacter sp;* **ST1-6** - *Uncultured bacterium;* **ST1-12** - *Enterobacteriaceae; bacterium,*
PT1-4 - *Staphylococcus aureus;* **PT1-6** - *Bacillus megaterium;* **PT5-1** - *Thiobacillus aquaesulis*

SI Figure S4. Biofilm formation assay (quantitative estimation of the biofilm was done by microtiter plate assay).

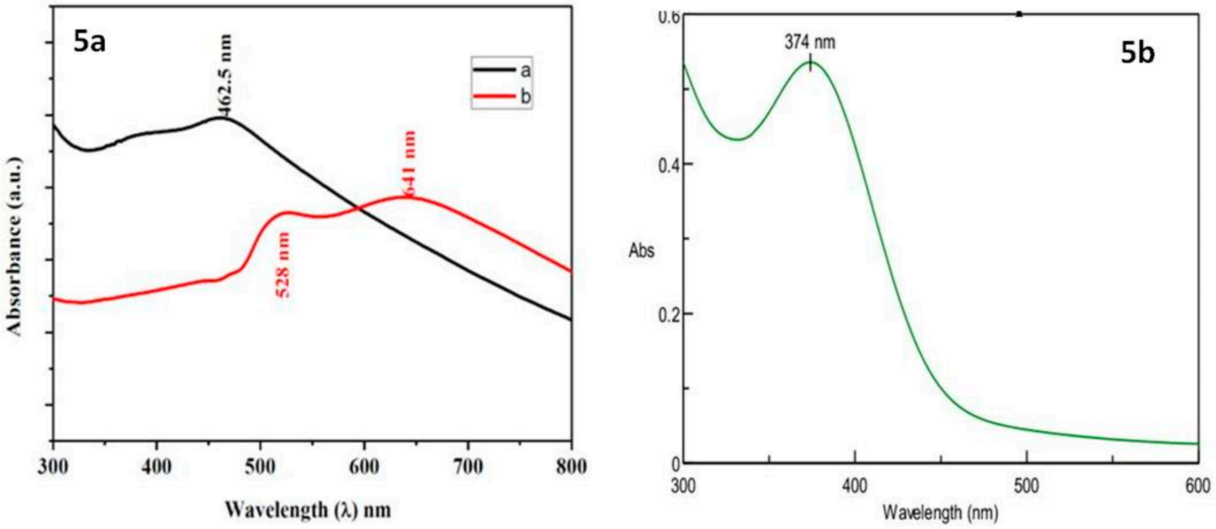

SI Figure S5. a) UV-Vis spectrum of Cu_2O and (**b**) UV-Vis spectrum of ZnO

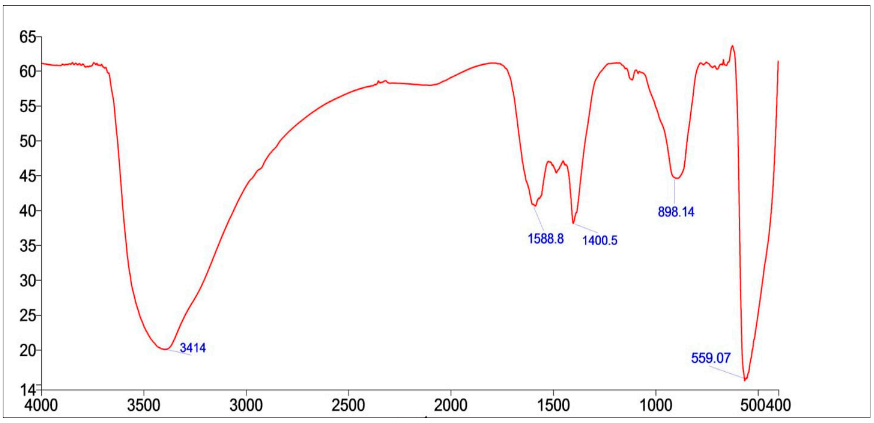

SI Figure S6a FTIR pattern of ZnO.

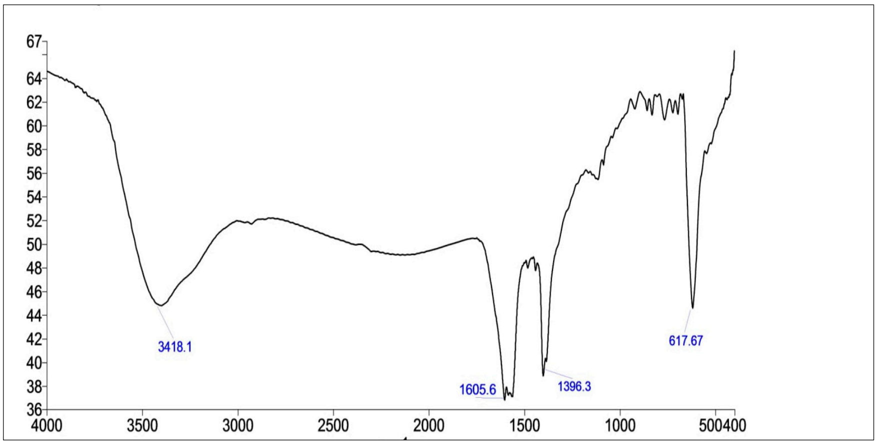

SI Figure S6b FTIR pattern of Cu_2O.

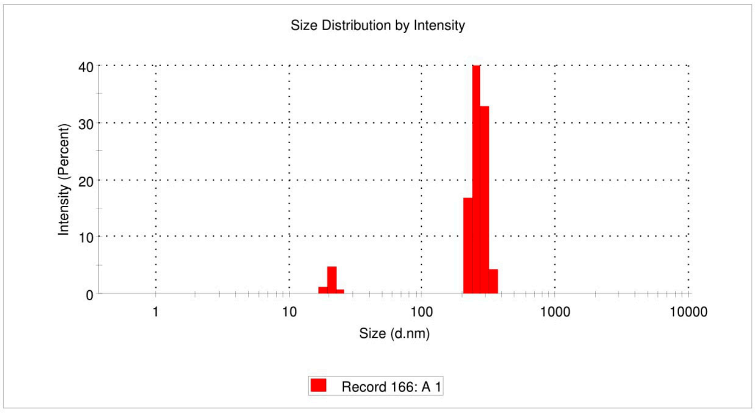

SI Figure S7a DLS result of ZnO.

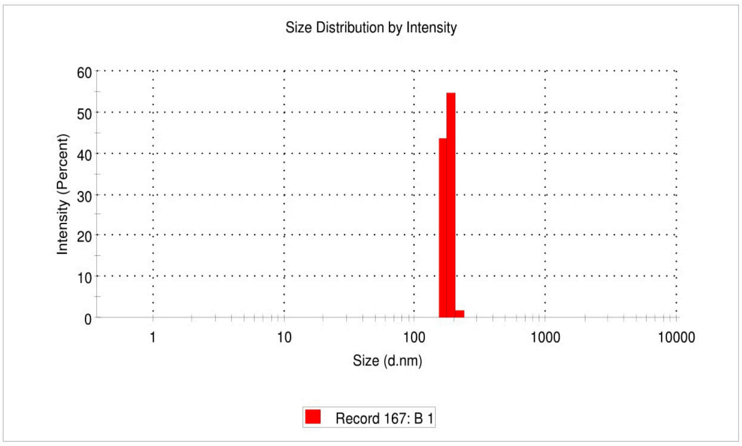

SI Figure S7b DLS result of Cu_2O.

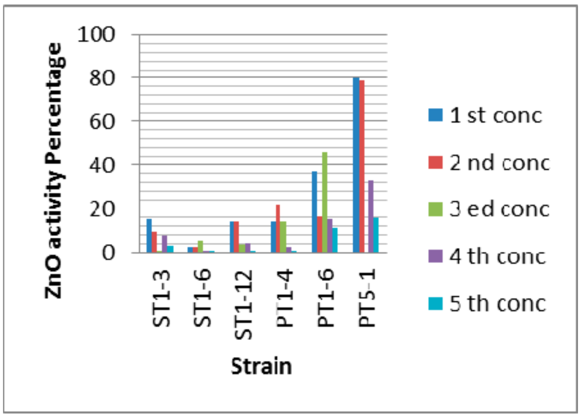

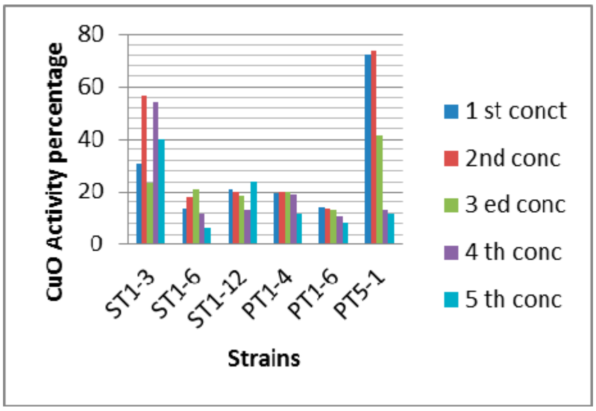

SI Figure S8 a,b: Different concentration of ZnO and Cu_2O nanoparticle activity (**ST1-3** *Acinetobacter sp,* **ST1-6** *Uncultured bacterium,* **ST1-12** *Enterobacteriaceaebacterium,* **PT1-4***Staphylococcus aureus,***PT1-6** *Bacillus megaterium,* **PT5-1** *Thiobacillus aquaesulis***).**

SI Table S1 Morphological and physiological characteristics of isolated bacterial strains

train name	Gram's staining	Spore staining	Capsule Staning	Oxidase Disc	Catalase	Indole	MR	VP	Citrate	Urease	TSI			
											Base	slant	H_2S	Gas
ST1-1	- rod	+	-	+	-	-	-	-	-	+	AL	AL	-	-
ST1-2	-rod	+	-	+	-	-	-	+	-	-	AL	AL	-	-
ST1-3	-cocci	+	-	+	-	+	+	+	-	+	AC	AC	-	+
ST1-4	-rod	-	-	+	+	+	+	-	-	-	AC	AL	-	-
ST1-5	-rod	+	-	-	+	+	-	+	-	-	AL	AL	-	-
ST1-6	- rod	+	-	-	+	+	+	-	-	+	AC	AC	-	+
ST1-7	-rod	+	-	-	+	+	+	-	-	+	AC	AC	-	+
ST1-8	- rod	-	-	+	-	-	+	-	-	+	AC	AC	-	+
ST1-9	- rod	+	-	+	-	-	+	+	+	+	AC	AC	-	+
ST1-10	- rod	+	-	+	+	+	+	-	+	+	AL	AC	+	-
ST1-11	- rod	+	-	-	+	+	+	-	+	+	AC	AC	-	+
ST1-12	- rod	+	-	+	+	+	+	+	+	+	AC	C	-	+
ST5-1	- rod	+	-	-	+	-	+	+	-	+	AC	AC	-	+
ST5-5	-rod	+	-	+	-	-	-	+	-	-	AL	AL	-	-
ST5-8	- rod	+	-	-	+	-	+	+	-	-	AL	AC	-	-
ST5-9	- rod	+	-	+	+	-	+	+	-	+	AL	AL	-	-
ST5-10	- rod	-	-	+	+	-	-	+	-	-	AL	AL	-	-
ST5-11	+cocci	+	-	+	+	-	+	+	+	+	AC	AC	-	+
PT1-1	+cocci	-	-	-	+	+	+	-	+	+	AC	AC	-	+
PT1-2	+rod	+	-	-	+	+	-	+	+	+	AC	AC	-	+
PT1-3	+cocci	+	-	-	+	+	+	-	+	+	AC	AC	-	+
PT1-4	+rod	+	-	-	+	+	+	-	+	+	AC	AC	-	+
PT1-5	+rod	+	-	-	+	-	-	-	+	+	AL	AL	-	-
PT1-6	+rod	+	-	-	+	-	-	-	+	+	AC	AL	-	+
PT5-1	- rod	+	-	-	+	+	-	-	+	+	AC	AC	-	+
PT5-2	+rod	-	-	-	-	+	+	-	+	+	AC	AC	-	+
PT5-3	- rod	+	-	-	+	+	+	-	+	+	AC	AC	-	+
